# Mycobacterial Lineages Associated with Drug Resistance in Patients with Extrapulmonary Tuberculosis in Addis Ababa, Ethiopia

**DOI:** 10.1155/2021/5239529

**Published:** 2021-09-18

**Authors:** Getu Diriba, Abebaw Kebede, Habteyes Hailu Tola, Ayinalem Alemu, Bazezew Yenew, Shewki Moga, Desalegn Addise, Zemedu Mohammed, Muluwork Getahun, Mengistu Fantahun, Mengistu Tadesse, Biniyam Dagne, Misikir Amare, Gebeyehu Assefa, Dessie Abera, Kassu Desta

**Affiliations:** ^1^Ethiopian Public Health Institute, Addis Ababa, Ethiopia; ^2^Department of Medical Laboratory Sciences, College of Health Sciences, Addis Ababa University, Addis Ababa, Ethiopia; ^3^Department of Microbial, Cellular and Molecular Biology, College of Natural and Computational Sciences, Addis Ababa University, Addis Ababa, Ethiopia; ^4^St. Paul's Hospital Millennium Medical College, Addis Ababa, Ethiopia; ^5^Armauer Hansen Research Institute, Addis Ababa, Ethiopia

## Abstract

**Background:**

In Ethiopia, tuberculosis (TB) is one of the most common causes of illness and death. However, there is limited information available on lineages associated with drug resistance among extrapulmonary tuberculosis patients in Ethiopia. In this study, researchers looked into *Mycobacterium tuberculosis* lineages linked to drug resistance in patients with extrapulmonary tuberculosis in Addis Ababa, Ethiopia.

**Methods:**

On 151 *Mycobacterium tuberculosis* isolates, a cross-sectional analysis was performed. Spoligotyping was used to characterize mycobacterial lineages, while a phenotypic drug susceptibility test was performed to determine the drug resistance pattern. Data were analyzed using SPSS version 23.

**Results:**

Among 151 *Mycobacterium tuberculosis* complex (MTBC) genotyped isolates, four lineages (L1–L4), and *Mycobacterium bovis* were identified. The predominantly identified lineage was Euro-American (73.5%) followed by East-African-Indian (19.2%). Any drug resistance (RR) and multidrug-resistant (MDR) tuberculosis was identified among 16.2% and 7.2% of the Euro-American lineage, respectively, while it was 30.8% and 15.4% among the East-African-Indian lineages. Among all three preextensively drug-resistance (pre-XDR) cases identified, two isolates belong to T3-ETH, and the other one strain was not defined by the database. There was no statistically significant association between any type of drug resistance and either lineage or sublineages of *Mycobacterium tuberculosis*.

**Conclusion:**

A higher proportion of any type of drug resistance and MDR was detected among the East-African-Indian lineage compared to others. However, there was no statistically significant association between any type of drug resistance and either lineages or sublineages. Thus, the authors recommend a large-scale study.

## 1. Introduction

Currently, tuberculosis (TB) is the first and the 10th global cause of death among infectious diseases and all diseases [1]. It is a global public health issue that affects approximately one-fourth of the world's population [1]. Based on the 2020 Global TB report, it is estimated that about 10 million people develop TB. Across the globe, there were 1.2 million TB deaths among HIV-negative people and 208, 000 deaths among HIV-positive people [1, 2]. The Mycobacterium tuberculosis complex (MTBC), the causative agent of TB, spreads from one person to the other through sneezing, coughing, and talking. Tuberculosis is mainly a disease of the lung (pulmonary TB), but it also affects other body parts (extrapulmonary TB) [1, 3].

Nowadays, TB control programs are challenged due to the emergence of drug-resistant TB and in particular multidrug-resistant (MDR) and extensively drug-resistance TB (XDR-TB) [4]. Globally, the burden of multidrug- or rifampicin-resistant TB (MDR/RR-TB) as a share of the number of TB cases remains stable. In 2019, an estimated 3.3% of new TB cases and 18% of previously treated cases had MDR/RRTB. In absolute numbers, there were an estimated 465, 000 incident cases of RR-TB burden [1, 5, 6]. Additionally, XDR-TB accounts for 7.4% of MDR-TB worldwide [5].

Ethiopia is among the 14 high TB, MDR-TB, and TB-HIV burden countries in the world. Based on the 2020 global TB report, the prevalence of MDR-TB among new and retreatment cases was reported to be 0.71% and 16%, respectively [1, 7]. Besides, the pre-XDR-TB and XDR-TB isolates accounted for 5.7% and 0.6%, respectively [8].

These nine MTBC genetic lineages are categories into modern lineages, intermediate lineage, and ancestral lineages. The “modern” clades of tuberculosis lineage are East Asian, East-African-Indian, and Euro-American lineages (lineages 2 and 3 and 4 and 7) and lineages 7 [9–12], whereas Indo-Oceanic (lineage 1), *Mycobacterium bovis*, and *Mycobacterium africanum* West African 1 and West African 2 (lineages 5 and 6) are ancestral lineages. The newly identified lineages 8 and 9 are an intermediate lineage [11, 12]. The East Asian and Euro-American lineages are the most common worldwide, and they are likely more virulent than other MTBC lineages [13]. The Latin American Mediterranean family and Euro-American (lineage 4) are the most common in Ethiopia [14]. A new lineage (lineage 7) has recently been identified, which is primarily found in Ethiopia and the Horn of Africa [15, 16].

A previous study conducted in the USA reported that the Euro-American, East Asian, Indo-Oceanic, and East African-Indian bacterial lineages were the predominantly identified bacterial lineages found in exclusively extrapulmonary tuberculosis patients (EPTB). Likewise, in exclusively PTB cases, the predominantly identified lineages were Euro-American, East Asian, Indo-Oceanic, and East African-Indian bacterial lineages [17]. In a given situation, characterization of those lineages in EPTB may provide better insights on strain pathogenicity and further will help to document global TB epidemiology [18]. Even though some published studies characterized the drug resistance pattern of MTBC in EPTB patients in Ethiopia [19], there is limited information on the association between specific mycobacterial lineages and sublineages with drug resistance in EPTB patients. Thus, the aim of this study was to identify the Mycobacterial lineages linked to drug resistance in extrapulmonary tuberculosis patients in Addis Ababa, Ethiopia.

## 2. Materials and Methods

### 2.1. Study Setting

In this study, we characterized about 151 MTBC isolates identified from 151 patients with extrapulmonary tuberculosis. The isolates were based on samples (abscess, ascetic fluid, CSF, lymph node aspirate, pleural fluid, peritoneal fluid, pericardium fluid, pus) collected from EPTB patients in the selected eight public hospitals located in Addis Ababa from 2016 to 2017. Drug-susceptibility testing and spoligotyping of Mycobacterial isolates are performed at the Ethiopian Public Health Institute. The laboratory provides TB research services, TB diagnosis, and drug susceptibility tests by well-experienced professionals using advanced technologies.

### 2.2. Study Design and Period

Primary data were collected in a period between December 2016 and August 2017, while the current laboratory-based cross-sectional study was conducted from November 2019 to April 2020 using the stored MTB isolates identified from extrapulmonary cases in Addis Ababa, Ethiopia.

### 2.3. Laboratory Tests

#### 2.3.1. Drug-Susceptibility Testing

All isolates were subjected to drug susceptibility testing (DST) with first-line anti-TB drugs streptomycin (STM; 1 g/ml), isoniazid (INH; 0.1 g/ml), rifampicin (RIF; 40 g/ml), ethambutol (EMB; 5 g/ml), and pyrazinamide (PZA; 100/ml) using the MGIT 960 [20]. Second-line DST was performed on multidrug-resistant isolates using second-line anti-TB drugs such as amikacin (AMK; 1.0 g/ml), capreomycin (CAP; 1.25 g/ml), ethionamide (ETH; 2.5 g/ml), kanamycin (KAN; 2.5 g/ml), moxifloxacin (MOX; 2.5 g/ml), and ofloxacin (OFX; 2.0 g/m [21]). Reference strain MTB H37Rv was used for quality control.

#### 2.3.2. Spoligotyping

Genomic DNA was extracted from grown subcultured isolates by transferring 500 *μ*l growth *Mycobacterium tuberculosis* isolate into 1.5 cryovial tubes, followed by heat inactivation for 20 minutes at 95°C and release the mycobacterial DNA. Recovered DNA was either immediately used for PCR amplification or stored at -20°C until use [22]. Spoligotyping was carried out according to the standard protocol developed by Kamerbeek et al. [23]. We run both positive and negative controls for each run. For the positive control, we used characterized strains of H37Rv and BCG strains. While for the negative control, molecular grade water is used. Hybridized PCR products were incubated with streptavidin peroxidase conjugate, and the membrane was exposed to a chemiluminescence system, followed by exposure to X-ray film. The film was then developed using standard photochemical procedures (Amersham International, Buckinghamshire, United Kingdom) [23]. Spoligotype international type (SIT) and the family assignment were performed using the MIRU-VNTR*plus* database.

#### 2.3.3. Data Analysis

The spoligotype data analysis was conducted using the MIRU-VNTRplus database software for strain identification [24]. The database is used to assign the spoligotype international type (SIT) and family assignment. All data that were collected are entered into a Microsoft Excel sheet and analyzed using SPSS version 23. Frequencies and proportions were determined to characterize the type of lineages, sublineages, and the DST pattern. Odds ratio along with the 95% CI was used to assess the association between the drug resistance pattern and the type of mycobacterial lineages and sublineages. A *P* value < 0.05 was considered as a statistically significant difference.

## 3. Results

### 3.1. Mycobacterial Lineages Identified

Among the 151 isolates, we identified four main lineages with nine sublineages. Euro-American strain (L4) was the most frequent lineage (111 isolates, 73.5%), followed by the East-African-India (L3) (26 isolates, 17.2%). At the sublineage level, the most prevalent sublineages identified were T family (47 isolates, 31.1%), followed by T3-ETH (33 isolates, 21.9%), Central Asia Strain (26 isolates, 17.2%), Haarlem (15 isolates, 9.9%), and unknown (nine isolates, 6.0%) **(**Table 1**)**.

### 3.2. *Mycobacterium tuberculosis* Lineages and their Drug Resistance Patterns

The spoligotype patterns and their associated drug resistance were analyzed for the 151 mycobacterial isolates analyzed in this study. Out of 151 isolates tested, 122 were sensitive to all first-line anti-TB drugs and belong to different lineages like Euro-American (L4, 61.6%), East-African-Indian (L3, 11.9%), East-Asian (L2, 0.7%), Bovis (1.3%), and unknown strain (5.4%). Among all 151 isolates, we observed 29 drug-resistant isolates. Among the Euro-American lineages (L4), the resistance pattern to first-line anti-TB drugs was as follows: STM (5, 4.5%), INH (14, 12.6%), RIF (8, 7.2%), EMB (2, 1.3%), PZA (14, 12.9%), and MDR-TB (8, 7.2%). Of the East-African-Indian lineages (L3), 3 (11.5%), 5 (19.2%), 4 (15.4%), 6 (23.1%), and 4 (15.4%) lineages became resistant to STR, INH, RIF, PZA, and MDR-TB, respectively. In the current study, only one isolate was identified as Indo-Oceanic (L1), and the isolate was resistant to STR, INH, and RIF. However, a single East-Asian (L2) Beijing isolate becomes susceptible to all drugs. Among the three *Mycobacterium bovis* identified, one strain becomes resistant to INH and PZA. MDR-TB was identified in 6.3% [7], 0.9% [1], 15.4% [4], 100% [1], and 11.1% [1] of T3-ETH, Haarlem, CAS, EAI5, and undefined mycobacterial sublineages, respectively (Table 2).

Of the 14 MDR-TB isolates that had also been submitted to DST for second-line DST (AMK, CAP, ETH, KAN, MOX, and OFX), only three pre-XDR cases were identified. Of these three isolates, two isolates that belong to T3-ETH became resistant to MOX and OFX. The other one strain, which is not defined strain in the database, becomes resistant to CAP and ETH (Table 3).

Any type of drug resistance was identified in 16.2% [18], 30.8% [8], and 11.1% [1] among the Euro-American lineage, East-African-Indian lineage, and unknown strains, respectively. However, in this study, a statistically significant association was not observed for Euro-American lineage (COR; 0.51, 95% CI, 0.216-1.204), East-African-Indian lineage (COR; 2.201, 95% CI, 0.846-5.724), other lineages (COR; 2.938, 95% CI, 0.468-18452), and the strain with undefined lineage (COR; 0.509, 95% CI, 0.61-4.238) with any type of drug resistance. Similarly, in subgroup analysis, there was no statistically significant association between the type of sublineage and any type of drug resistance (Table 4).

## 4. Discussion

The present study describes the circulating MTBC lineages and the associated drug-resistance pattern among MTBC isolates collected from EPTB patients. In Ethiopia, few studies have been performed using extrapulmonary samples, and the MTBC lineages associated with drug resistance are poorly described in the country. According to the results of the current analysis, the Euro-American lineage (L 4) was the most prevalent (73.5%), followed by the East-African-Indian (L3) lineage (19.2%). Additionally, we showed that the T family strain predominates (31.1%), followed by T3-ETH (21.9%), Central Asia Strain (17.2%), Haarlem (9.9%), and *Mycobacterium bovis* (2%). A higher proportion of drug resistance was identified among the East-African-Indian lineage (30.8%), followed by the Euro-American lineage (16.2%). However, this study revealed that there was no statistically significant association between any type of drug resistance and either the type of mycobacterial lineage or sublineage.

In the present study, Indo-Oceanic lineage, East-African-Indian lineage, East-Asian lineage, and the Euro-American lineage were identified. Lineage 4 (Euro-American lineage) was the most prevalent among the lineages listed. The dominant prevalence of lineage 4 (Euro-American) and lineage 3 (East-African-Indian) has already been shown in previous studies in Ethiopia [14, 25, 26]. The Euro-American lineage was found to be the most prevalent lineage in Ethiopia and the United States, confirming our results [15, 17, 27–29]. Likewise, a previously conducted study revealed that the T family that is categorized under Euro-American was predominant [30]. In Sudan, MTBC lineage 4 (the Euro-American lineage) was also the most common (71.6%) [29]. Previously conducted studies in India also identified 76.05%, 67.5, and 59.3% of Euro-American strains [31–33]. The Euro-American lineage's widespread distribution may be due to its virulence and population movement between areas, which promotes its transmission.

Drug resistance to each first-line anti-TB drug, including STM, INH, RMP, EMB, and PZA, was observed to be more prevalent in the EAI lineage and the Euro-American among MTBC genotypes in this study, although not statistically significant. Previous studies in Ethiopia have linked MTBC of the Euro-American lineage with resistance to first-line anti-TB drugs [34, 35]. In our study, East-African-Indian lineage in CAS sublineage was found to be driving the massive spread of drug resistance TB to compare the other than strains.

In the present study, a higher prevalence of MDR was found among the CAS-Delhi/East-African-Indian (lineage 3) followed by the T3-ETH/Euro-American lineage (lineage 4). Likewise, previous studies conducted in Ethiopia reported a higher prevalence of MDR among T3-ETH and CAS-Delhi/East-African-Indian MTB*C* strains that were isolated from lymph node aspirates [36] .

Resistance to INH was the most frequent in the Euro-American (L4) lineage with predominantly the T3-ETH sublineage. Likewise, the INH mono-resistance was predominantly identified in the T3-ETH sublineage. Previous studies from Ethiopia conducted using direct spoligotyping have also reported similar detection rates of INH mono-resistant isolates belonged to diverse families within Euro-American (L4) [37]. As a consequence, it is necessary to develop a closer surveillance system in the country, with a particular emphasis on isolates where resistance to isoniazid is observed, and to reduce the evolution of DR to MDR and subsequent dispersion within the population.

In the current study, XDR-TB was not identified, despite the fact that 25% of MDR-TB patients of the Euro-American (L4) had pre-XDR-TB cases. In addition, the most frequent resistance proportion was observed in the T3-ETH sublineage. However, we were unable to compare our data to that of other studies in the country because there was no data on pre-XDR/XDR Mycobacterium tuberculosis resistance isolates in combination with EPTB genotypes. However, the finding is in agreement with a previous investigation in Mexico where the Euro-American lineage was predominantly identified among the pre-XDR/XDR *Mycobacterium tuberculosis* isolates [38]. More importantly, the higher prevalence rate of MDR-TB strains with additional resistance to the second-line anti-TB drugs in this study warrants the urgent need for reinforcement of the TB control program [28]. Finally, this study has some limitations. First, the sample size was relatively small. Secondly, the study was based on the mycobacterial isolates collected from EPTB patients residing in a single city, Addis Ababa, which might not represent the whole country.

## 5. Conclusions

In conclusion, MTBC strains showed considerable genetic diversity in Addis Ababa, Ethiopia. Euro-American (L4) with T3-ETH sublineage strain family and the East-African-Indian (L3) in CAS sublineages were the dominant lineages in both the total MTBC and drug-resistance MTBC strains. No statistically significant association was observed between any type of drug resistance with MTBC lineage or sublineages. Thus, a study on a larger number of MTBC isolates and comparing from both PTB and EPTB is needed to clearly describe the epidemiology of MTBC lineages associated with drug resistance in extrapulmonary tuberculosis patients.

## Figures and Tables

**Table 1:** Distribution of *Mycobacterium tuberculosis* complex (MTBC) lineages and families among extrapulmonary TB isolates (*n* = 151).

Lineages (L)	Sublineages	Frequency	Percent
Euro-American (L4)		111	73.5%
	T	47	31.1%
	T3-ETH	33	21.9%
	Haarlem	15	9.9%
	X1	1	0.7%
	LAM	6	4.0%
	Ural	6	4.0%
East-African-Indian (L3)	CAS	26	17.2%
Indo-Oceanic (L1)	EAI5	1	0.7%
East-Asian (L2)	Beijing	1	0.7%
Bovis	BOV	3	2.0%
Unknown strain	Not defined	9	5.3%

EAI: East-African-Indian; IO: Indo-Oceanic; EAI: East-African-Indian; CAS: Central Asian Strain; H: Harlem; BOV: Bovis; LAM: Latin American Mediterranean; ETH: Ethiopia; L: lineages.

**Table 2:** Distribution of first-line drug-resistance with *Mycobacterium tuberculosis* complex lineages and families among extrapulmonary TB (*N* = 151).

Lineages (Ls) (*n*)	Sublineages	Resistance *N* (%)					
		STR	INH	RIF	EMB	PZA	MDR-TB
Euro-American (L4) (111)		5 (4.5)	14 (12.6)	8 (7.2)	2 (1.8)	14 (12.6)	8 (7.2)
	T	1 (0.9)	2 (1.8)	0	1 (0.9)	2 (1.8)	0
	T3-ETH	3 (2.7)	10 (9.0)	7 (6.3)	1 (0.9)	0	7 (6.3)
	Haarlem	1 (0.9)	1 (0.9)	1 (0.9)	0	3 (2.7)	1 (0.9)
	X1	0	0	0	0	0	0
	LAM	0	1 (0.9)	0	0	2 (1.8)	0
	Ural	0	0	0	0	1 (0.9)	0
East-African-Indian (L3) [26]	CAS	3 (11.5)	5 (19.2)	4 (15.4)	0	6 (23.1)	4 (15.4)
Indo-Oceanic (L1) [1]	EAI5	1 (100)	1 (100)	1 (100)	0	0	1 (100)
East-Asian (L2) [1]	Beijing	0	0	0	0	0	0
Bovis [3]	BOV	0	1 (33.3)	0	0	1 (33.3)	0
Unknown strain [9]	Not defined	0	1 (11.1)	1 (11.1)	1 (11.0)	1 (11.1)	1 (11.1)

EA: Euro-American; EAI: East-African-Indian; IO: Indo-Oceanic; EAI: East-African-Indian; CAS: Central Asian Strain; H: Harlem; BOV: Bovis; LAM: Latin American Mediterranean; ETH: Ethiopia; L: lineages; STM: streptomycin; INH: isoniazid; RIF: rifampicin; EMB: ethambutol; PZA: pyrazinamide; MDR: multidrug resistance.

**Table 3:** Distribution of second-line anti-TB drug-resistance with lineages and families among MDR isolates among EPTB cases (*N* = 14).

Lineages (Ls) (*n*)	Sublineages	Resistance *N* (%)						
		AMK	CAP	ETH	KAN	MOX	OFX	Pre-XDR-TB
Euro-American (L4) [8]								
	T3-ETH	0	0	0	0	2 (25.0)	2 (25.0)	2 (25.0)
Unknown strain [1]	Not defined	0	1 (100)	1 (100)	0	0	0	1 (100)

ETH: ethinomidine; L: lineages; OFX: ofloxacin; CAP: capreomycin; AMK: amikacin; KAN: kanamycin; MOX: moxifloxacin; ETH: ethionamide.

**Table 4:** Association between any drug resistance and lineages and sublineage genotype of *Mycobacterium tuberculosis* among extrapulmonary TB isolates (*n* = 151).

Characteristic	Variable	Any drug resistance				
		Sensitive	Resistant	Total	COR (95% CI)	*P*
Lineages (L)	Euro-American	93	18	111	0.51 (0.216-1.204)	0.124
	East-African-Indian	18	8	26	2.201 (0.846-5.724)	0.106
	Unknown strain	8	1	9	0.509 (0.61-4.238)	0.532
	Others lineages	3	2	5	2.938 (0.468-18452)	0.250
Sublineages	T	43	4	47	0.294 (0.096-0.900)	0.032
	T3-ETH	24	9	33	1.837 (0.744-4.540)	0.187
	Haarlem	13	2	15	0.621 (0.132-2.918)	0.546
	LAM	4	2	6	2.185 (0.380-12.551)	0.381
	Ural	5	1	6	0.949 (0.102-8.824)	0.963
	CAS	21	5	26	1.943 (0.755-4.998)	0.168
	Unknown strain	8	1	9	0.509 (0.61-4.238)	0.532
	Other sublineages	4	2	6	1.965 (0.236-16.363)	0.532

EAI: East-African-Indian; CAS: Central Asian Strain; H: Harlem; LAM: Latin American-Mediterranean; ETH: Ethiopia; L: lineages; CI: confidence interval; COR: crude odds ratio; other lineages: Indo-Oceanic, East-Asian, and Bovis; other sublineages: EAI5, Beijing, and Bovis.

## Data Availability

The datasets used and/or analyzed during the current study are available from the corresponding author upon reasonable request.

## Abbreviations

AMK: Amikacin
BOV: Bovis
CAP: Capreomycin
CAS: Central Asian Strain
CI: Confidence interval
COR: Crude odds ratio
EA: Euro-American
EAI: East-African-Indian
IO: Indo-Oceanic
EAI: East-African-Indian
EMB: Ethambutol
ETH: Ethiopia
H: Harlem
INH: Isoniazid
KAN: Kanamycin
L: Lineages
LAM: Latin American Mediterranean
MDR: Multidrug resistance
MOX: Moxifloxacin
MTBC: Mycobacterium tuberculosis complex
OFX: Ofloxacin
PZA: Pyrazinamide
RIF: Rifampicin
STM: Streptomycin
TB: Tuberculosis
XDR: Extensively drug-resistance.
